# Soil Microbial Networks Shift Across a High-Elevation Successional Gradient

**DOI:** 10.3389/fmicb.2019.02887

**Published:** 2019-12-18

**Authors:** Emily C. Farrer, Dorota L. Porazinska, Marko J. Spasojevic, Andrew J. King, Clifton P. Bueno de Mesquita, Samuel A. Sartwell, Jane G. Smith, Caitlin T. White, Steven K. Schmidt, Katharine N. Suding

**Affiliations:** ^1^Department of Ecology and Evolutionary Biology, Tulane University, New Orleans, LA, United States; ^2^Institute of Arctic and Alpine Research, University of Colorado Boulder, Boulder, CO, United States; ^3^Department of Entomology and Nematology, University of Florida, Gainesville, FL, United States; ^4^Department of Evolution, Ecology, and Organismal Biology, University of California, Riverside, Riverside, CA, United States; ^5^Biosciences Division, Oak Ridge National Laboratory, Oak Ridge, TN, United States; ^6^King Ecological Consulting, Knoxville, TN, United States; ^7^Department of Ecology and Evolutionary Biology, University of Colorado Boulder, Boulder, CO, United States

**Keywords:** bacteria, diversity, fungi, interaction network, joint distribution model, 16S, 18S, ITS

## Abstract

While it is well established that microbial composition and diversity shift along environmental gradients, how interactions among microbes change is poorly understood. Here, we tested how community structure and species interactions among diverse groups of soil microbes (bacteria, fungi, non-fungal eukaryotes) change across a fundamental ecological gradient, succession. Our study system is a high-elevation alpine ecosystem that exhibits variability in successional stage due to topography and harsh environmental conditions. We used hierarchical Bayesian joint distribution modeling to remove the influence of environmental covariates on species distributions and generated interaction networks using the residual species-to-species variance-covariance matrix. We hypothesized that as ecological succession proceeds, diversity will increase, species composition will change, and soil microbial networks will become more complex. As expected, we found that diversity of most taxonomic groups increased over succession, and species composition changed considerably. Interestingly, and contrary to our hypothesis, interaction networks became less complex over succession (fewer interactions per taxon). Interactions between photosynthetic microbes and any other organism became less frequent over the gradient, whereas interactions between plants or soil microfauna and any other organism were more abundant in late succession. Results demonstrate that patterns in diversity and composition do not necessarily relate to patterns in network complexity and suggest that network analyses provide new insight into the ecology of highly diverse, microscopic communities.

## Introduction

Microbes are important regulators of ecosystem function, and much research has been directed at testing how soil microbial composition and diversity shift along environmental gradients. Work, thus far, has identified many important abiotic and biotic drivers that structure the composition and diversity of microorganisms in the soil, including pH, salinity, climate, soil nutrients, plant diversity, and plant functional traits (Fierer and Jackson, 2006; Lozupone and Knight, 2007; Nemergut et al., 2010; de Vries et al., 2012; Porazinska et al., 2018). However, how *interactions* among microbial taxa change across the landscape is much less well understood (Nemergut et al., 2013). Previous studies have used microbial co-occurrence patterns to construct networks and make inferences about microbial interactions (Barberán et al., 2012; Widder et al., 2014; Williams et al., 2014; Morriën et al., 2017; de Vries et al., 2018); however, these methods are limited because microbial co-occurrence can reflect both species interactions (e.g., predation, facilitation, decomposition) and shared environmental niches (co-occurrence due to shared preference for abiotic conditions). Recent advances in hierarchical joint species distribution modeling allow us to parse out the effect of environmental variables to better capture interactions *per se* (Hui and Poisot, 2016; Ovaskainen et al., 2017); yet they have rarely been applied to microbial datasets (Collins et al., 2018). Here we used this technique to test whether patterns in the complexity of bacterial and eukaryotic networks follow patterns in diversity and compositional change across a fundamental gradient, ecological succession.

While the vast majority of research on community patterns along successional gradients has focused on macroorganisms, shifts in microbial communities are beginning to be addressed. Recent work suggests that the composition of microbes changes considerably and predictably during primary succession (Nemergut et al., 2007; Tarlera et al., 2008; Roy-Bolduc et al., 2015; Poosakkannu et al., 2017), with certain groups like Actinobacteria and N fixers prevalent in early successional stages, and other groups such as Acidobacteria and mycorrhizal taxa dominating later stages (Rime et al., 2015; Poosakkannu et al., 2017; Yarwood and Högberg, 2017). Much work also suggests that the taxonomic diversity (Nemergut et al., 2007; Tarlera et al., 2008; Brown and Jumpponen, 2014; Rime et al., 2015; Poosakkannu et al., 2017) and functional diversity (Tscherko et al., 2003) of soil bacterial and fungal communities generally increase during succession. However, some studies find decreases, no change, or variable patterns for some or all taxa (Sigler and Zeyer, 2002; Brown and Jumpponen, 2014; Dini-Andreote et al., 2014; Poosakkannu et al., 2017).

While patterns in diversity and composition of microbial communities during succession are beginning to emerge, virtually nothing is known about how microbial networks shift across that same gradient. The network structure of communities, independent of diversity and composition, has important implications for resilience (Welti and Joern, 2015; Mandakovic et al., 2018), stability (Neutel et al., 2007), and the efficiency of carbon transfer in an ecosystem (D’Alelio et al., 2016; Morriën et al., 2017). Studies of macroorganisms generally find that interaction networks increase in complexity across succession. In pollination biology (Albrecht et al., 2010; Losapio et al., 2015) and work on animal food webs (Wardle et al., 1995; Neutel et al., 2007), network complexity (typically measured as the number of connections per species) increases due to the increase in quantity and diversity of resources over succession. Experiments have also shown that substrate limitation can constrain the complexity of animal food webs (Chen and Wise, 1999). This same reasoning should hold true for microbial food webs and interaction networks over succession (Bardgett et al., 2005), but it has rarely been studied (but see Dini-Andreote et al., 2014). The diversity of carbon substrates increases over succession (Hooper et al., 2000; Nemergut et al., 2007; Milcu and Manning, 2011). More carbon substrates mean a higher diversity of microorganisms to degrade those substrates and more mutualistic or predatory relationships among taxa can arise (Boer et al., 2005). Furthermore, microbial communities often act in consortia to synergistically degrade complex plant-derived compounds, with some microbes utilizing metabolites or taking advantage of breakdown products of extracellular enzymes produced by other taxa (Lynd et al., 2002; Alessi et al., 2017). Thus, due to the increases in carbon quantity and diversity over succession, we expected the complexity of microbial interactions to increase as well.

Here we combined classical community measures of diversity and species composition with measures of putative species interaction networks using hierarchical Bayesian joint species distribution modeling to ask how microbial community structure changes across a high elevation successional gradient. Our system is a high-elevation alpine landscape in which local variation in environmental conditions (e.g., topography, snow depth) results in strong differences in successional stage across space, as measured by differences in a suite of ecological variables including plant cover, plant diversity, soil nutrients, organic matter content, and microbial biomass. We investigated bacterial and eukaryotic communities, including single-celled eukaryotes, fungi, and soil microfauna, as well as plant communities, to capture multitrophic interactions among core soil organisms. Incorporating multiple taxonomic groups is notable as most soil microbial network research has been limited to a single domain of life, primarily bacteria, and thus has not captured higher-order predation and grazing food webs (but see Steele et al., 2011; Morriën et al., 2017). We hypothesized that over the successional gradient, diversity will increase, species composition will change, and soil microbial networks will become more complex.

## Materials and Methods

### Study Site

Our study site is a south-facing subnival slope in Green Lakes Valley Watershed at Niwot Ridge LTER (40°3′24″N 105°37′30″W, Supplementary Figure S1). It is approximately 2 km^2^ in area, ranging between 3610 and 3940 m in elevation, and is composed of talus block slopes, late-melting snowbanks covering unvegetated gravel soils, fellfields, and patches of tundra vegetation. Plant cover ranges from 0% in late-melting snowbanks up to ∼75% (170 individuals m^–2^) in exposed areas. The site is covered with snow from October to June and the deepest snowfields do not melt fully until August or September. This site was previously sampled in 2007 (King et al., 2010, 2012); we report here on a sampling done in 2015.

Sampling locations were based on a grid in which most plots were ∼50 m apart, and in three targeted sampling areas where plots were 5 m apart (King et al., 2010) (Supplementary Figure S1). We sampled 98 plots that were circular with 1 m radius. Soil samples, for microbial community analysis and biogeochemistry, were collected September 8–17, 2015, 1–2 weeks after the main snowbeds melted out. Three subsamples of soil (∼3 cm diameter, 4 cm depth) were taken from each plot and combined. Samples were homogenized in a ziplock bag, and subsamples for sequencing and microfauna extraction were weighed out the same day and stored at −20 and 4°C, respectively, until processing. Samples were then stored overnight at 4°C, and subsampled for gravimetric soil moisture, water holding capacity (WHC), inorganic N, and total C (TC) and N (TN), microbial C and N, and pH.

Vegetation was surveyed during peak biomass, August 19–September 3, 2015. Plant density was measured by counting all plants within the 3.14 m^2^ circle and ranged from 0 to 169 plants/m^2^. Clonal plants and cushion plants were counted as clumps, such that counts represent our best estimate of single genets. Moss was also counted as clumps. Lichen was not included in the plant dataset, because the fungi and algae/cyanobacteria that compose it are measured in the microbe datasets. Plant cover was measured using the point intercept method, assessing the presence of vegetation, rock, and bare soil at 40 points within the plot.

### Laboratory Molecular Methods

We chose metabarcoding gene markers specific to each microbial group of interest: 16S for bacteria, ITS for fungi, and 18S for eukaryotes. For eukaryotes, we designated two groups: (1) small, often single-celled, eukaryotes (protists and algae), and (2) soil “microfauna” (small invertebrates including nematodes, tardigrades, rotifers, platyhelminthes, small arthropods, and small annelids). All groups except the microfauna were extracted directly from 0.35 g soil. Because this volume is too small to accurately capture abundance of microfauna, they were first extracted from ∼20 g soil subsamples using Whitehead trays (Porazinska et al., 2014). Briefly, each tray (20 cm × 20 cm × 5 cm) was equipped with a supporting sieve lined with a thin tissue. Soil was spread evenly over the tissue, wetted with 150 ml of water, and left to extract at room temperature for 24 h. Water from each tray was passed through a 38 μm mesh sieve, captured microfauna were transferred to 15 ml sterile Falcon tubes, and allowed to settle overnight at 4°C. The volume was reduced to ∼0.5 ml with sterile disposable pipettes and transferred to bead beating tubes from the PowerSoil DNA isolation kit (MOBIO Laboratories Inc., Carlsbad, CA, United States).

All DNA was extracted using a PowerSoil DNA Isolation Kit according to the manufacture’s protocol. Each sample was PCR amplified twice using primers (515F/806R, ITS1-F/ITS2, and 1391f/EukBr), multiplexing barcodes (Goley), and conditions as adapted by the Earth Microbiome Project^1^ (Amaral-Zettler et al., 2009; Caporaso et al., 2012). Amplicons were purified and normalized with SequalPrep Normalization Kits (Invitrogen Inc., CA, United States), combined into three single pools of 16S, ITS, and 18S amplicon libraries, and sequenced on three lanes using Illumina technology (MiSeq2000, pair-end 2 × 300 bp) at the BioFrontiers Institute, Boulder, CO, United States.

### Sequence Analysis

The raw read sequence data were processed using amplicon sequence variants (ASV) methods in QIIME2 version 2018.2 (Callahan et al., 2017). We demultiplexed and trimmed primers and adapters in QIIME2 and denoised the data and joined paired reads using DADA2. For bacteria and ITS reads that showed poor quality at the ends, we truncated reads where median quality score fell below ∼30; however, DADA2 is robust to low-quality sequence because it incorporates read quality information into its error model. We assigned taxonomy using a pre-trained Naive Bayes classifier. The classifier was trained on the Greengenes 13.8 database (DeSantis et al., 2006) for 16S, UNITE 7.2 (Abarenkov et al., 2010) for ITS, and SILVA-ARB release 111 (Pruesse et al., 2007) for 18S. 16S and 18S sequences were aligned using mafft, and trees were built using FastTree within QIIME2.

### Biogeochemistry

We measured a number of environmental variables to characterize the successional stage of each plot and to use as explanatory variables in the joint distribution models. Gravimetric soil moisture was measured on 5 g of soil by drying for 48 h at 60°C. WHC was determined by placing 4 g soil in 15 ml bottom-meshed conical tubes, saturating the soil and allowing to drain, weighing the saturated soil, and then drying at 60°C for 15 h and weighing the dried soil.

Microbial biomass N and C were determined using the chloroform fumigation method (Robertson et al., 1999). Briefly, a 5 g subsample of soil was extracted immediately with 0.5 M K_2_SO_4_ and another subsample was fumigated with chloroform to kill microbes and then extracted. Total dissolved nitrogen (TDN) and dissolved organic carbon (DOC) were then analyzed using a Shimadzu total organic carbon analyzer equipped with a TDN module (Shimadzu Scientific Instruments, Inc., Columbia, MD, United States), and microbial biomass N and C were calculated as the difference between the fumigated and unfumigated samples. Inorganic N (NH_4_^+^, NO_3_^–^) was measured on the unfumigated extracts with a Lachat QuickChem 8500 Flow Injection Analyzer (Lachat Instruments, Loveland, CO, United States) and Synergy 2 Multi-Detection Microplate Reader (BioTek Instruments, Inc., Vinooski, VT, United States).

Total C and TN were measured on 4 g air-dried soil, ground manually with mortar and pestle, and analyzed with a Thermo Finnigan Flash EA 1112 Series CHN analyzer (Thermo Fisher Scientific, Inc.). To measure pH, 2 g of soil was suspended in 3 ml of ultrapure water (Honeywell) and shaken for 1 h, and pH was recorded when the reading stabilized using a calibrated Oakton benchtop pH meter (Oakton Instruments, Vernon Hills, IL, United States).

Snow depth in each plot was calculated from annual snow depth surveys conducted in the Green Lakes Valley from 1997 to 2015 (Losleben, 2002; Morse, 2019). Surveys occur in May at peak snowpack along a grid of random points (mean *n* = 483) spaced ∼50 m apart. We used kriging interpolation to create a continuous raster surface for each year, conferred snow depth to each plot, and then averaged over the 19 years to reflect long-term snow cover conditions. We also calculated the coefficient of variation (CV) in snow depth for each plot (standard deviation/mean) to quantify variability in snow depth. Elevation (m. a. s. l.) was recorded using a handheld Trimble GPS device with an error rate of 3 m.

### Statistical Analysis

Our final data set consisted of the 75 plots that had sufficient sequencing depth shared across all organism groups and available data for all biogeochemical analyses. Prior to analyses, all data sets were rarefied to an even sampling depth (bacteria rarefied to 7987, fungi to 1023 small eukaryote to 871, microfauna to 700). We recognize that this sampling depth is relatively shallow; however, our network analysis focuses on abundant species, thus we believe we are capturing relevant community members (see Supplementary Figure S2 for rarefactions). Prior to network construction, microbial taxa were classified as photosynthetic, heterotrophic, chemoautotrophic, or unknown using a number of taxonomic references (Maddison and Schulz, 2007; Rosenberg et al., 2014a,b,c,d,e,f; Horton et al., 2019). All analyses were done in R (R Core Team, 2017).

We classified plots as early, mid, or late succession by running a PCA of environmental and plant variables previously determined to change over successional gradients in alpine areas (Bekku et al., 2004; Schmidt et al., 2008; Porazinska et al., 2018) (TC, TN, NH_4_^3+^, NO_3_^–^, microbial biomass C, microbial biomass N, pH, WHC, soil moisture, snow depth, elevation, plant density, plant Shannon diversity, and plant cover) using the vegan package (Oksanen et al., 2016) (see Supplementary Figure S3 for the PCA and Supplementary Table S1 for pairwise Pearson correlations). We chose to include all variables in the PCA because succession is associated with changes in all variables; however, results are robust to the choice of variables used to define successional stage. Axis 1 of the PCA explained 50.9% of the variation and was used as a proxy for successional stage (Axis 2 only explained 14.8% of the variation so was not used). Plot scores were extracted and divided into three groups of 25 corresponding to early, mid, and late succession (see Supplementary Figure S1 for photos of plots). We recognize that succession in this system is a continual process and splitting the data into three groups with equal number of plots is not ideal. However, it was necessary to split groups evenly (25 plots in each successional stage) so that sample size would not influence the number of microbial taxa being modeled and the statistical power to detect significant relationships. We chose to split data into *three* groups, because a sample size of *n* = 25 plots in each successional stage was reasonable based on the complexity of (df needed for) the joint distribution modeling described below.

To compare diversity of bacteria, fungi, small eukaryotes, and microfauna across succession, Faith’s phylogenetic diversity, ASV richness (Chao1), Pielou’s evenness, rarity, and frequency were calculated. Phylogenetic diversity was calculated using the package picante (Kembel et al., 2010) in R for all groups except fungi, because the fungal ITS sequenced region is highly variable and difficult to align (Moore and Frazer, 2007). Rarity was calculated as the proportion of taxa with relative abundances less than 1/S (Camargo, 1992), where S is the mean ASV richness. Frequency was calculated as the number of plots (out of 25) in which each taxon was present in early, mid, and late successional stages. To test the effect of successional stage on all diversity metrics except frequency, ANOVAs were performed using the gls() function in package nlme (Pinheiro et al., 2019) in R accounting for spatial autocorrelation with a spherical autocorrelation structure. To test the effect of successional stage on frequency, a quasi-poisson regression was performed using the glm() function in R (autocorrelation was not included since the observational unit is taxon rather than plot, quasipoisson models were preferred due to slight overdispersion). Differences among successional stages were tested using Tukey *post hoc* tests with glht() from the multcomp package (Hothorn et al., 2008).

Compositional shifts across the successional gradient were assessed for each taxonomic group using ordination techniques and visualized using relative abundance barplots and krona plots. For ordination, because microbiome datasets are compositional (relative abundance) and thus have a negative correlation bias, the centered log-ratio (clr) transformation was used (Gloor et al., 2017). We used cmultRepl() in package zCompositions to impute zeros (estimate a small non-zero number for the zeros, because you cannot take the log of zero), and then calculated clr. Redundancy analysis (RDA) was performed on the clr transformed data (also known as the Aitchison distance) (Gloor et al., 2017), and permutation tests were done to test the effect of successional stage on community composition using R package vegan (Oksanen et al., 2019). To visualize compositional shifts over succession at a higher taxonomic level, we calculated relative abundance of different groups of bacteria (phylum level), fungi (phylum level), small eukaryotes (phylum or major clade level, much of the taxonomy is not resolved), and microfauna (phylum level), separating photosynthetic, chemoautotrophic, and heterotrophic taxa within the groups. The effect of successional stage on the most abundant groups was tested using linear models including spatial autocorrelation structure (spherical model), and ANOVAs were performed with *P*-values adjusted to control the false discovery rate (Benjamini and Hochberg, 1995). Krona interactive plots were also created (Ondov et al., 2011) using the psadd package (Pauvert, 2019) in R and are available in FigShare (Farrer, 2019).

Hierarchical Bayesian joint species distribution modeling was performed using the package boral (Hui and Poisot, 2016) in R. We used clr transformed data to reduce negative correlation bias, and we only included ASVs that occurred in at least 12 plots (out of 25) in each successional stage; thus, we suggest that this is a “core” microbial network focused on interactions among frequent taxa. We fit the joint distribution model with three latent variables and included autocorrelation structure (spherical model) to account for non-independence due to spatial arrangement of plots. We use forward selection to select the four environmental explanatory variables that explained the most variation in our models (snow depth, pH, soil moisture, and CV snow depth), with the realization that many environmental variables were correlated with one another. We used actual environmental variables (as opposed to Axis 1 of the PCA which we used to define successional stage), because we wanted more dimensionality to our analysis and we wanted our results to be generalizable to other systems. For early, mid, and late successional models, there was some correlation among the four environmental explanatory variables (seven out of 18 pairs were correlated: two in early, two in mid, and three in late); however, the absolute values of all correlation coefficients were ≤0.62. When models were run including only environmental variables and autocorrelation structure, those four variables explained an average of 27% of the variance in ASV (clr) abundance, suggesting that it is important to remove their effect. We fit the model using MCMC with 40,000 iterations, a burn-in of 10,000, and a thin of 30. Convergence was checked using Geweke diagnostics and trace plots, and model fit was assessed using Dunn–Smyth residuals and a normal quantile plot (see Supplementary Figure S4). We calculated the residual species-to-species correlation matrix, which represents species correlations after accounting for the effects of the environmental variables. We interpret these correlations as representing species interactions; however, they may also represent unmeasured environmental gradients, which is discussed further in the discussion.

We built networks based on correlations whose 95% posterior credible interval did not overlap zero. Networks were visualized using the package igraph (Csardi and Nepusz, 2006). We calculated linkage density as our metric of complexity (average number of edges per node), and we tabulated number of nodes (ASVs), number of edges (connections between the nodes), number of positive and negative correlations, and the number of bacteria, fungi, small eukaryotes, and soil microfauna included in each network. All code can be found on GitHub: https://github.com/ecfarrer/NWT_MovingUphill5.

### Randomizations and Network Assessment

Prior studies have shown that species richness can affect the number of network connections (Faust et al., 2015), and our data show that microbial richness increases across succession. We performed four tests to ensure that the observed patterns of network complexity over the successional gradient were real and not due to statistical artifacts.

First, we assessed our false-positive rate in the networks, because microbe–microbe connections in the network may be due to random chance. We randomized our early, mid, and late successional datasets (*n* = 10 randomizations for each successional stage) using randomizeMatrix() in picante (Kembel et al., 2010) using the method that randomizes abundances within taxa and maintains species occurrence frequency. Then we ran the joint distribution modeling analysis on this randomized data, assessed the number of significant network connections, and converted this to a percent by dividing by the number of network connections in models using our observed data.

Second, we did simulations to test the effect of taxonomic richness on the number of network connections. Our datasets had two properties that we wanted to preserve in our simulated data, (1) our late successional plots had the highest taxonomic richness, but (2) despite high richness, after applying our frequency cutoff (only modeling species that were present in ≥12 plots, see above), the late succession dataset had fewer taxa that were included in joint distribution modeling compared to early and mid succession datasets (in other words, most of the diversity in late successional plots comprised infrequent taxa). Therefore, we simulated count datasets that varied in species richness by drawing from the probabilities (relative abundances) of the bacterial data from our early and late successional plots. We used a Dirichlet–multinomial distribution using the rmultinom() function and the rdirichlet() function from the MCMCpack package (Martin et al., 2011) as in Faust et al. (2015). For each simulation set, we simulated 25 samples with 2000 reads. We performed joint distribution modeling on these simulated data using only latent variables and assessed the number of significant network connections. Then we repeated the simulations 25 times each for “early” and “late” successional stages. This generated a dataset (*n* = 50) with variability in mean total taxonomic richness and variability in the richness of frequent species (passing our ≥ 12 frequency cutoff). We assessed the effect of species richness and number of taxa used in modeling on number of network connections and network complexity using linear regression.

Third, because our early, mid, and late successional datasets had different numbers of taxa that passed our frequency cutoff (306, 301, and 273, respectively) and were included in distribution modeling, we subsampled our data to make sure that the network complexity patterns were robust to the number of taxa in the analysis. For the early and mid successional plots, we randomly chose 273 taxa out of the 306 and 301 taxa, respectively, and performed distribution modeling and assessed network complexity (*n* = 10 times for each successional stage).

Fourth, because the PCA (Supplementary Figure S3) showed that the late successional plots comprised a greater range in variability compared to early or mid successional plots, we did subsetting and modeling to ensure that this did not affect our results. We chose a similar range in PCA Axis 1 (approximately 0.35 Axis 1 units), and randomly chose 12 plots from early, mid, and late successional categories within that range (12 plots was the greatest number of late successional plots that could be obtained within that range). We performed distribution modeling and assessed network complexity for these three reduced models.

## Results

### Diversity and Composition

**FIGURE 1:**
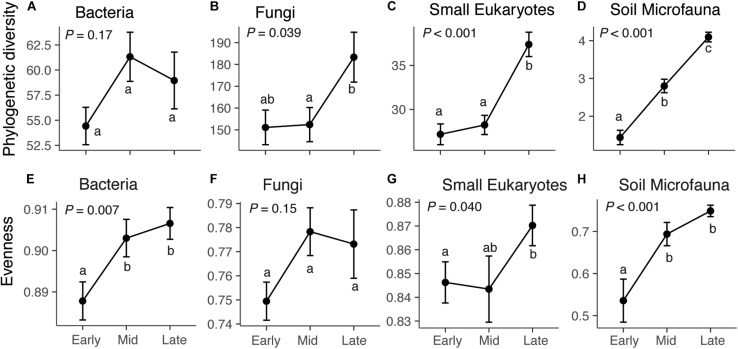
Diversity and evenness of microbial communities across succession: bacteria **(A,E)**, fungi **(B,F)**, small eukaryotes **(C,G)**, and soil microfauna **(D,H)**. For bacteria, small eukaryotes, and soil microfauna, the diversity metric is Faith’s phylogenetic diversity, which is a measure of taxonomic richness. For Fungi, the metric is ASV richness (Chao1), since the ITS region cannot be aligned to give phylogenetic information. Successional stage does not have a significant effect on bacterial diversity (*F*_2_,_72_ = 1.82, *P* = 0.17), but does affect fungi (*F*_2_,_72_ = 3.41, *P* = 0.039), small eukaryotes (*F*_2_,_72_ = 21.01, *P* < 0.001), and soil microfauna (*F*_2_,_72_ = 54.23, *P* < 0.001). Successional stage affects evenness of bacteria (*F*_2_,_72_ = 5.28, *P* = 0.007), small Eukaryotes (*F*_2_,_72_ = 3.36, *P* = 0.040), and microfauna (*F*_2_,_72_ = 10.65, *P* < 0.001), but not fungi (*F*_2_,_72_ = 1.96, *P* = 0.15). Results of Tukey *post hoc* tests for comparing multiple treatments are shown as letters. Values shown are means and standard errors. Note that the *y*-axis scales are different for each taxonomic group.

Across our microbial datasets, phylogenetic diversity of small eukaryotes and microfauna increased over succession (Figure 1). ASV richness of fungi, small eukaryotes, and microfauna increased over succession (Figure 1 and Supplementary Figure S5), and taxonomic evenness of bacteria, small Eukaryotes, and microfauna increased over succession (Figure 1). The proportion of rare (low abundance) taxa was highest in late succession for small Eukaryotes and soil microfauna, but did not change over succession for bacteria and fungi (Supplementary Figure S5). The average frequency (number of plots) in which taxa were present decreased over succession for bacteria, fungi, and small Eukaryotes; however, the opposite trend was present in the soil microfauna (Supplementary Figure S5).

**FIGURE 2:**
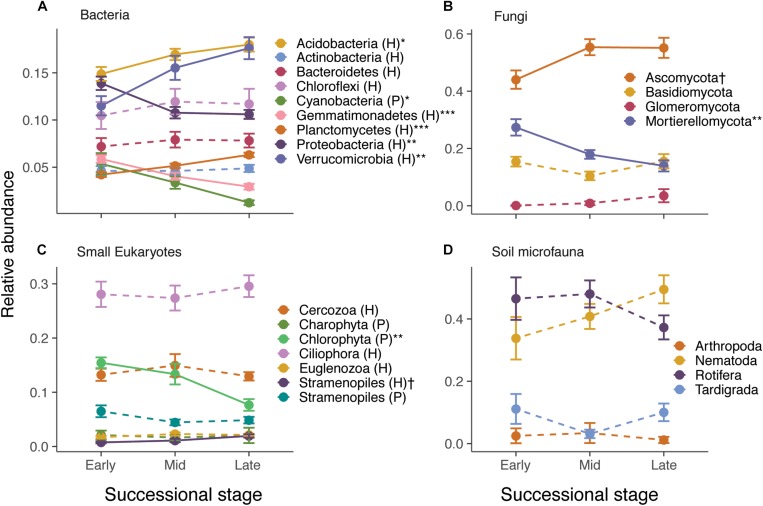
Effect of successional stage on the relative abundance of major taxonomic groups of bacteria **(A)**, fungi **(B)**, small eukaryotes **(C)**, and soil microfauna **(D)**. Note that only abundant groups are shown in the panels. P and H refer to photosynthetic and heterotrophic groups, respectively. The effect of successional stage on each of the groups was tested using separate anovas, with *P*-values corrected for false discovery rate using Benjamini–Hochberg: ^†^*P* < 0.10, ^∗^*P* < 0.05, ^∗∗^*P* < 0.01, ^∗∗∗^*P* < 0.001. Solid lines indicate significant or nearly significant relationships and dashed lines indicate non-significant relationships.

Species composition also changed considerably across the gradient. RDA ordinations showed that successional stage explained 8.2–10.2% (*P* < 0.001) of the variance in ASV composition of bacteria, fungi, small eukaryote, and soil microfauna communities (Table 1 and Supplementary Figure S6). Relative abundance of some photosynthetic bacteria and eukaryotes (Cyanobacteria, Chlorophyta) decreased over succession (Figure 2). Heterotrophic members of the Verrucomicrobia, Planctomycetes, and Stramenopiles increased in abundance. The Ascomycota increased over succession, whereas the Mortierellomycota decreased by about half (Figure 2B). No significant patterns were seen at the phylum level for the soil microfauna (Figure 2D).

**TABLE 1:** Permutational multivariate analysis of variance testing the effect of successional stage on community composition for the four microbial groups.

	**Variance explained**	**Pseudo-*F***	***P***
Bacteria	9.3%	3.71	<0.001
Fungi	10.2%	4.09	<0.001
Small eukaryotes	8.2%	3.21	<0.001
Soil microfauna	8.2%	3.20	<0.001

*For ordination biplots, see Supplementary Figure S6.*

### Microbial Networks

**FIGURE 3:**
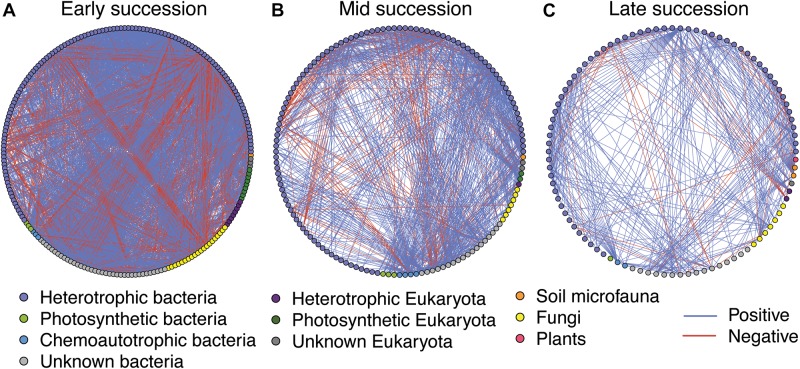
Microbial networks across a successional gradient from early **(A)**, mid **(B)**, and late **(C)** successional stages. Interactions were assessed using the residual species-to-species correlation matrix from a hierarchical joint distribution model, which removes the effect of environmental covariates on species distributions. Displayed here are the correlations whose 95% credible intervals did not overlap zero. Nodes are colored based on taxonomy. See Table 2 for network statistics.

Microbial networks decreased in complexity across the successional gradient (Figure 3 and Table 2). Complexity (measured as linkage density, the average number of connections per taxon) decreased from 16.7 to 6.9 to 4.7. Both the number of taxa involved in the networks and the number of correlations decreased over succession (Table 2). The majority of network interactions were positive, and the percentage of positive interactions was highest in late succession (early: 77.9%, mid: 82.0%, late: 88.9%). The number of photosynthetic microbes in networks decreased over succession (Table 2). Soil microfauna increased slightly in networks over succession, with one taxon present in early and mid successional networks and two in the late successional network. Plants were present only once in the networks, in late succession (the grass *Trisetum spicatum*).

**TABLE 2:** Network statistics for microbial networks across a successional gradient (for the network diagrams, see Figure 3).

	**Early**	**Mid**	**Late**
	**succession**	**succession**	**succession**
Linkage density (complexity)	16.7	6.9	4.7
Nodes (taxa)	209	140	96
Edges (connections)	3481	963	452
% Positive interactions	77.9%	82.0%	88.9%
# Bacteria	168	127	83
# Fungi	20	7	7
# Small eukaryotes	20	5	3
# Soil microfauna	1	1	2
# Plants	0	0	1
# Photosynthetic microbes	12	5	1

*Linkage density (complexity) is the average number of edges per node. Nodes are the number of taxa in each network. Edges are the connections (correlations) between the nodes.*

Multiple tests showed that our networks were robust and that the observed pattern of increasing taxonomic diversity but decreasing network complexity over succession is not due to statistical artifacts. False positive rates in our networks were very low: networks of randomized data had an average of 1.0 (±0.5), 3.1 (±1.1), and 2.9 (± 0.9) significant microbe–microbe connections for early, mid, and late succession networks, respectively, which suggests 0.029, 0.32, and 0.64% of the interactions in our actual networks may be false positives. Performing joint distribution modeling on simulated datasets with varying taxonomic richness showed that neither the total richness nor the richness of frequent species (those passing our ≥ 12 frequency cutoff used in distribution modeling) affected the number of significant network connections (*R*^2^ = 0.0088, *P* = 0.24, *R*^2^ = 0.026, *P* = 0.14, respectively) or network complexity (*R*^2^ = 0.021, *P* = 0.53, *R*^2^ = 0.013, *P* = 0.27, respectively). Furthermore, when we controlled for the number of taxa passing the frequency cutoff by subsetting the early and mid successional datasets so that the same number of species was included in joint distribution modeling as the late successional dataset, the pattern of reduced complexity across the successional gradient held: the total number of network connections was still higher in early and mid successional areas compared to late (2699 ± 48, 774 ± 20 vs. 452) as was the network complexity (number of interactions per taxon, 14.6 ± 0.2, 6.2 ± 0.1, vs. 4.7). Lastly, when we controlled for the range of PCA axis 1 by running models on reduced datasets in which early, mid, and late successional plots all comprised the same spread along PCA axis 1 (0.35 units each), we found that the pattern of decreasing complexity across the gradient held as well: the total number of connections (678, 156, 60) and the network complexity (10.9, 5.4, 2.9) decreased across the gradient in these reduced but standardized datasets.

## Discussion

Testing how different metrics of microbial community structure shift across the landscape is an important step in understanding the complex, yet unseen, majority that regulates ecosystem function. Consistent with our hypothesis, we found that microbial diversity generally increased across the successional gradient and species composition shifted in ways that benefited certain groups (heterotrophic Verrucomicrobia, Ascomycota, heterotrophic Stramenopiles) over others (Cyanobacteria, Mortierellomycota, Chlorophyta). However, contrary to our expectations, the complexity (defined as the average number of interactions per taxon) of core microbial networks (of frequent taxa) decreased across the successional gradient. Results highlight a surprising complexity of photosynthetic and heterotrophic microbial interactions in sparsely vegetated soil and suggest that different analytical metrics (diversity vs. microbial interactions) may yield different conclusions about the complexity of microbial communities.

It is commonly found that microbial diversity increases (Nemergut et al., 2007; Cline and Zak, 2015) and microbial composition changes (Nemergut et al., 2007; Dini-Andreote et al., 2014) over succession. We found that the diversity of fungi, small eukaryotes, and soil microfauna (but not bacteria) increases over succession, and we observed marked changes in composition in all taxonomic groups. The lack of (significant) increase in bacterial diversity is somewhat surprising, but previous work has shown that bacteria dominate the active community of unvegetated soils during the summer months at this site (Ley and Schmidt, 2002) likely because they have a much broader range of physiologies (e.g., chemoautotrophy, N-fixation) than do fungi and other eukaryotes (Schmidt et al., 2014). Increases in eukaryotic microbial diversity are likely due, in part, to increases in resource availability (Waldrop et al., 2006), such that a greater number of taxa are able to meet minimum resource requirements as resources increase. Overall, the harsh environmental conditions (low nutrients, low moisture) in early successional areas may limit microbes in the same way they limit plant growth. Also, over successional gradients, the number of complex carbon molecules in the system increases – saccharides, cellulose, phenolics, lignins, tannins – and one of the main controls on microbial diversity is the diversity of carbon substrates (Zhou et al., 2002). The shifting carbon inputs and changing abiotic conditions are also likely driving the compositional shifts observed. Some of the changes in relative abundance observed here agree with findings from other studies, such as an increase in Verrucomicrobia (Nemergut et al., 2007) and Acidobacteria (Nemergut et al., 2007; Yarwood and Högberg, 2017) over succession and an abundance of cyanobacteria (Schmidt et al., 2008; Zumsteg et al., 2012; Yarwood and Högberg, 2017) and algae (Kaštovská et al., 2005) in early successional stages. However, some of our results differ from prior studies that show decreases in Actinobacteria (Zumsteg et al., 2012; Poosakkannu et al., 2017) and shifts from Ascomycota to Basiodiomycota (Zumsteg et al., 2012) with succession in alpine and arctic areas, suggesting that succession may proceed differently at different sites even at a broad taxonomic level.

Despite general increases in diversity, we found that core microbial interaction networks decreased in complexity across the successional gradient. This same pattern was observed along a salt marsh chronosequence, where early successional stages displayed more network complexity (Dini-Andreote et al., 2014). However, other studies show that microbial network complexity increases over primary succession in forests (Yarwood and Högberg, 2017) and secondary succession in old fields (Morriën et al., 2017). Furthermore, studies show that carbon inputs increase network complexity during the short-term development of rhizosphere communities (Shi et al., 2016) and in a CO_2_ enrichment experiment (Zhou et al., 2011; Tu et al., 2015). All of these studies (with the exception of Morriën et al., 2017) focus only on one taxonomic group (bacteria or fungi), so they do not capture higher order trophic interactions and cascades.

In our system, one explanation for the decrease in network complexity over succession is the shift in primary producers and carbon resources across the gradient, from a dominance of algal/cyanobacterial and recalcitrant aeolian carbon inputs early in succession to a dominance of labile carbon compounds from root exudates as plant density increases. Our results show that early successional soils contain abundant photosynthetic bacteria and eukaryotes including Cyanobacteria, Chorophyta, and Stramenopiles (e.g., Diatomea and Chrysophyceae), and previous work at our study site has measured significant microbial carbon fixation in these barren, plant-free soils (Freeman et al., 2009b). These primary producers could form the base of food chains and interact with heterotrophs that feed on algal exudates, senescent algae, and algal detritus (Cole, 1982). In fact, studies in other systems show that the chemical constituents of algal exudates differ by species (Aluwihare and Repeta, 1999) and this affects the composition of bacterial heterotrophs (Kalscheur et al., 2012). Interactions between heterotrophic organisms and algae and cyanobacteria would result in high network complexity in early succession that would decline in later succession as increasing plant cover shades out photosynthetic microbes. This is borne out in the networks: the number of interactions between (frequent) bacterial heterotrophs and (frequent) photoautotrophic microbes declines over succession from 205 (early) to 55 (mid) to 1 (late). Additionally, the fourth most highly connected microbial taxon (potentially a type of “hub”) in the early successional networks was a photoautotrophic microbe in the Chloroflexi, whereas all highly connected taxa in mid and late successional networks were non-photosynthetic.

Further work at our study site shows that aeolian-deposited plant litter, particularly windblown pollen grains, is another important carbon source in early succession (Freeman et al., 2009a; Naff et al., 2013). Data from our study site indicate that barren talus soils contain a higher proportion of taxa that mineralize complex organic matter, like pollen, compared to vegetated soils (Ley et al., 2001). The breakdown of recalcitrant organic matter can involve numerous microbial taxa in decomposition, as bacteria that degrade complex substrates provide resources for bacteria that feed on metabolic byproducts or take advantage of monomers released by extracellular enzymes (Rakoff-Nahoum et al., 2014; Datta et al., 2016). This could result in complex interaction networks in early succession that would disappear as more labile plant root exudates dominate the system; however, this has not been experimentally tested. In our early succession networks, we found numerous positive interactions (146) between heterotrophic bacteria and members of the Ktedonobacteria (Phylum Chloroflexi), a group that is known to be important in degrading complex polymers, like pollen (Hug et al., 2013; King and King, 2014), and the number of heterotrophic bacteria–Ktedonobacteria interactions decrease over succession (33 in mid succession, four in late succession). Interestingly, the relative abundance of Ktedonobacteria does not change over succession, just their involvement in networks.

A major limitation in this study, and many studies of soil microbial communities, is the limited functional information about microbial taxa. This makes interpretation of network patterns across succession difficult. We took a first step by classifying taxa by mode of nutrition (photosynthetic/chemoautotrophic/heterotrophic) and by looking at patterns in groups for which we have information in our system (e.g., Ktedonobacteria). However, many interactions in our networks remain unexplained because we do not know the function of one or both of the interacting taxa (for 37% of the interactions, we do not even know if interacting organisms are photosynthetic or heterotrophic). As more functional information becomes available, it may open up new hypotheses explaining the observed drop in interaction complexity over succession.

### Network Interactions

Although we know very little about many of the taxa in the networks, we can highlight some interactions present in the network as proof of concept that the networks display realistic microbial relationships. In late successional networks, the grass *Tristum spicatum* was positively associated with a bacterium in the Sinobacteraceae, which has been shown to be a root associate of other plants (Tanaka et al., 2018). *Trisetum* was also positively associated with the Archaean, *Candidatus Nitrososphaera*, an ammonia oxidizing prokaryote (the first step in nitrification) (Rosenberg et al., 2014f). Archaeans are increasingly recognized as important plant symbionts especially in alpine areas (Taffner et al., 2018), and evidence suggests that *Trisetum* tends to prefer nitrate uptake to ammonium (Miller and Bowman, 2002). In late successional communities, we found relationships (positive and negative) between a bacterial-feeding nematode in the Plectidae (Yeates et al., 1993) and 28 different bacteria; both positive and negative relationships could be suggestive of consumption, depending on whether the predator limits prey availability or is limited by prey availability (Halliwell and Macdonald, 1996). Similarly, in early successional networks, we found relationships between a predatory nematode in the Nygolaimidae (Yeates et al., 1993) and 31 different eukaryotes, bacteria, and fungi. We also found positive correlations in mid succession communities between a bacterial feeding Heterolobosean amoeba (Brown et al., 2012; Pánek and Cepicka, 2012) and 15 bacterial taxa. And in early successional communities, we found correlations among two filter-feeding (subclass Scuticociliatia and Hypotrichia) ciliates and 41 different bacteria, which likely indicate feeding relationships (Lee and Capriulo, 1990; Jurgens and Simek, 2000). Overall, despite the lack of functional information for many of the microbial taxa in our networks, the interactions discussed here suggest that the methods used produce interpretable results.

### Limitations

While the hierarchical joint distribution modeling yielded realistic microbial relationships, had low false positive rates, and was not highly influenced by species richness like other correlational approaches (Faust et al., 2015), there are limitations. As with any correlation-based analysis, a key limitation in this work is that we cannot definitively conclude that all correlations represent interactions. While we attempted to isolate interactions *per se* by using hierarchical joint distribution modeling and removing the effect of environmental covariates, the residual correlations could represent shared niches that were not parceled out by the four chosen environmental covariates. For example, in early succession, there were five positive correlations among photosynthetic microbes (bacteria and algae), which might indicate that they share the same high light niche. Similarly, we did not include specific carbon substrates in the joint distribution model, so correlations among heterotrophic microbes may indicate shared niches specializing on a particular substrate (e.g., chitin, cellulose). We suggest that the putative interactions discovered by combining joint distribution modeling with network analysis be taken as hypotheses, which can be tested with future studies.

Our network analysis, by statistical necessity, only focuses on interactions among core, frequent microbial taxa. The analysis does capture a fairly wide range of taxon abundance: for example, for bacteria in early succession, the average relative abundance of taxa in the network ranged two orders of magnitude from 0.00051 to 0.057, and species with higher relative abundance were no more likely to be included in the network (i.e., have interactions with other microbes) vs. not. That said, there were many infrequent taxa that we could not attempt to model: in early succession, there were a total of 8442 taxa (bacteria, fungi, small eukaryotes, soil microfauna, plants) in the plots, but only 306 were frequent enough (present in > 11 plots) to be considered in the joint distribution modeling. It is difficult to predict how interactions with infrequent taxa would change network structure. Work on plants suggests that rare species may be facilitated more than dominant species (Choler et al., 2001); however, predation may have the opposite pattern (rare species may escape predation) (Murdoch and Oaten, 1975). Understanding the functioning of the rare biosphere is one of the major challenges in microbial ecology today, and new technologies and analytical methods are being developed to probe these rare community members (Jia et al., 2018).

Integrating diversity, species composition, and interaction networks gives us a comprehensive assessment of how systems change across succession at the community level; however, our methods do not address how ecosystem properties shift across succession. While there is a large literature on the link between biodiversity and ecosystem function (e.g., Hooper et al., 2005), there is scant information on the impact of network complexity on ecosystem level properties. One previous study showed that microbial network complexity is associated with increased efficiency of carbon uptake (Morriën et al., 2017), and by attempting to isolate interactions *per se*, our work provides some understanding of energy flow through an ecosystem since many interactions represent trophic relationships. The implications of network complexity for nutrient cycling and ecosystem function are a topic that is ripe for future studies.

## Conclusion

Microbes control many key aspects of ecosystem function, and ecologists have well documented the changes in diversity and composition of microbial communities across environmental gradients. However, less is known about how the network structure of microbes shifts across the landscape, and whether patterns in the complexity of species interactions follow patterns in complexity of diversity. Here we find that diversity increases across a successional gradient but network complexity declines. While much work remains to be done regarding the interpretation and implications of ecological network structure, using hierarchical joint distribution modeling to generate interaction networks is an important first step toward elucidating potential species interactions and important microbial players. The more we learn about the organization of microbial communities, the more we can leverage them for the many ecosystem services they provide.

## Data Availability Statement

The raw 16S, ITS, and 18S sequence data were deposited in the NCBI Sequence Read Archive (SRA): PRJNA587150.

## Author Contributions

EF, DP, MS, AK, KS, and SKS conceived the study. EF, DP, CB, SAS, JS, and CW collected field data and samples. EF, DP, CB, SAS, and CW performed laboratory biogeochemistry and molecular analyses. EF performed bioinformatics and data analysis and wrote the manuscript. All authors edited the manuscript and approved the final version of the manuscript.

## Conflict of Interest

AK was employed by Ecological Consulting (Knoxville, TN, United States). The remaining authors declare that the research was conducted in the absence of any commercial or financial relationships that could be construed as a potential conflict of interest.

## Footnotes

1 http://www.earthmicrobiome.org/emp-standard-protocols/
