# Supplementary material for: Soil Microbial Networks Shift Across a High-Elevation Successional Gradient
Source: Front Microbiol. 2019 Dec 18;10:2887. doi: 10.3389/fmicb.2019.02887 (PMC6930148; doi:10.3389/fmicb.2019.02887)
Supplement: Supplementary file 1 [file Table_1.DOCX]

Supplementary Material

Table S1. Pairwise correlations among variables included in the PCA used to define successional stage, and also including snow depth in 2015 and CV of snow which were included as potential explanatory variables in the joint distribution modeling.

|  | TC | TN | NH4 | NO3 | MicC | MicN | pH | WHC | Moisture | Snow depth | Elevation | Plant density | Plant diversity | Plant cover | Snow depth 2015 | CV snow depth |
| --- | --- | --- | --- | --- | --- | --- | --- | --- | --- | --- | --- | --- | --- | --- | --- | --- |
| TC | 1 | 0.98 | 0.57 | -0.04 | 0.84 | 0.81 | 0.22 | 0.88 | 0.68 | -0.13 | -0.26 | 0.63 | 0.52 | 0.52 | -0.01 | 0.14 |
| TN | 0.98 | 1 | 0.59 | 0.02 | 0.78 | 0.77 | 0.2 | 0.83 | 0.65 | -0.11 | -0.22 | 0.62 | 0.5 | 0.48 | 0.01 | 0.14 |
| NH4 | 0.57 | 0.59 | 1 | 0 | 0.46 | 0.51 | 0.17 | 0.42 | 0.37 | -0.03 | -0.26 | 0.33 | 0.29 | 0.2 | -0.05 | 0.11 |
| NO3 | -0.04 | 0.02 | 0 | 1 | -0.3 | -0.21 | -0.43 | -0.16 | -0.09 | 0.12 | 0.36 | -0.25 | -0.27 | -0.21 | 0.05 | -0.15 |
| MicC | 0.84 | 0.78 | 0.46 | -0.3 | 1 | 0.9 | 0.29 | 0.89 | 0.74 | -0.21 | -0.3 | 0.7 | 0.59 | 0.58 | -0.04 | 0.22 |
| MicN | 0.81 | 0.77 | 0.51 | -0.21 | 0.9 | 1 | 0.27 | 0.8 | 0.75 | -0.24 | -0.23 | 0.65 | 0.51 | 0.53 | -0.03 | 0.15 |
| pH | 0.22 | 0.2 | 0.17 | -0.43 | 0.29 | 0.27 | 1 | 0.24 | 0.11 | -0.52 | -0.43 | 0.5 | 0.65 | 0.37 | -0.34 | 0.35 |
| WHC | 0.88 | 0.83 | 0.42 | -0.16 | 0.89 | 0.8 | 0.24 | 1 | 0.7 | -0.14 | -0.33 | 0.68 | 0.58 | 0.57 | 0 | 0.18 |
| Moisture | 0.68 | 0.65 | 0.37 | -0.09 | 0.74 | 0.75 | 0.11 | 0.7 | 1 | -0.07 | -0.11 | 0.41 | 0.31 | 0.49 | 0 | 0.04 |
| Snow depth | -0.13 | -0.11 | -0.03 | 0.12 | -0.21 | -0.24 | -0.52 | -0.14 | -0.07 | 1 | -0.21 | -0.41 | -0.51 | -0.32 | 0.58 | -0.6 |
| Elevation | -0.26 | -0.22 | -0.26 | 0.36 | -0.3 | -0.23 | -0.43 | -0.33 | -0.11 | -0.21 | 1 | -0.26 | -0.26 | -0.11 | -0.12 | 0.12 |
| Plant density | 0.63 | 0.62 | 0.33 | -0.25 | 0.7 | 0.65 | 0.5 | 0.68 | 0.41 | -0.41 | -0.26 | 1 | 0.88 | 0.71 | -0.15 | 0.41 |
| Plant diversity | 0.52 | 0.5 | 0.29 | -0.27 | 0.59 | 0.51 | 0.65 | 0.58 | 0.31 | -0.51 | -0.26 | 0.88 | 1 | 0.72 | -0.25 | 0.47 |
| Plant cover | 0.52 | 0.48 | 0.2 | -0.21 | 0.58 | 0.53 | 0.37 | 0.57 | 0.49 | -0.32 | -0.11 | 0.71 | 0.72 | 1 | -0.11 | 0.19 |
| Snow depth 2015 | -0.01 | 0.01 | -0.05 | 0.05 | -0.04 | -0.03 | -0.34 | 0 | 0 | 0.58 | -0.12 | -0.15 | -0.25 | -0.11 | 1 | -0.4 |
| CV snow depth | 0.14 | 0.14 | 0.11 | -0.15 | 0.22 | 0.15 | 0.35 | 0.18 | 0.04 | -0.6 | 0.12 | 0.41 | 0.47 | 0.19 | -0.4 | 1 |

Fig. S1. Map of the 75 sampling plots with insets showing examples of early and late succession plots in the subnival zone of the Green Lakes Valley at Niwot Ridge LTER, Colorado, USA. The study area is 2 km from northeast corner to northwest corner. The plots are a subset of the plots in our previous studies (King *et al.* 2010; King *et al.* 2012) and were sampled in 2015. The scarcity of plots in the middle of the study area is due to a large cliff face with no soil or plants.


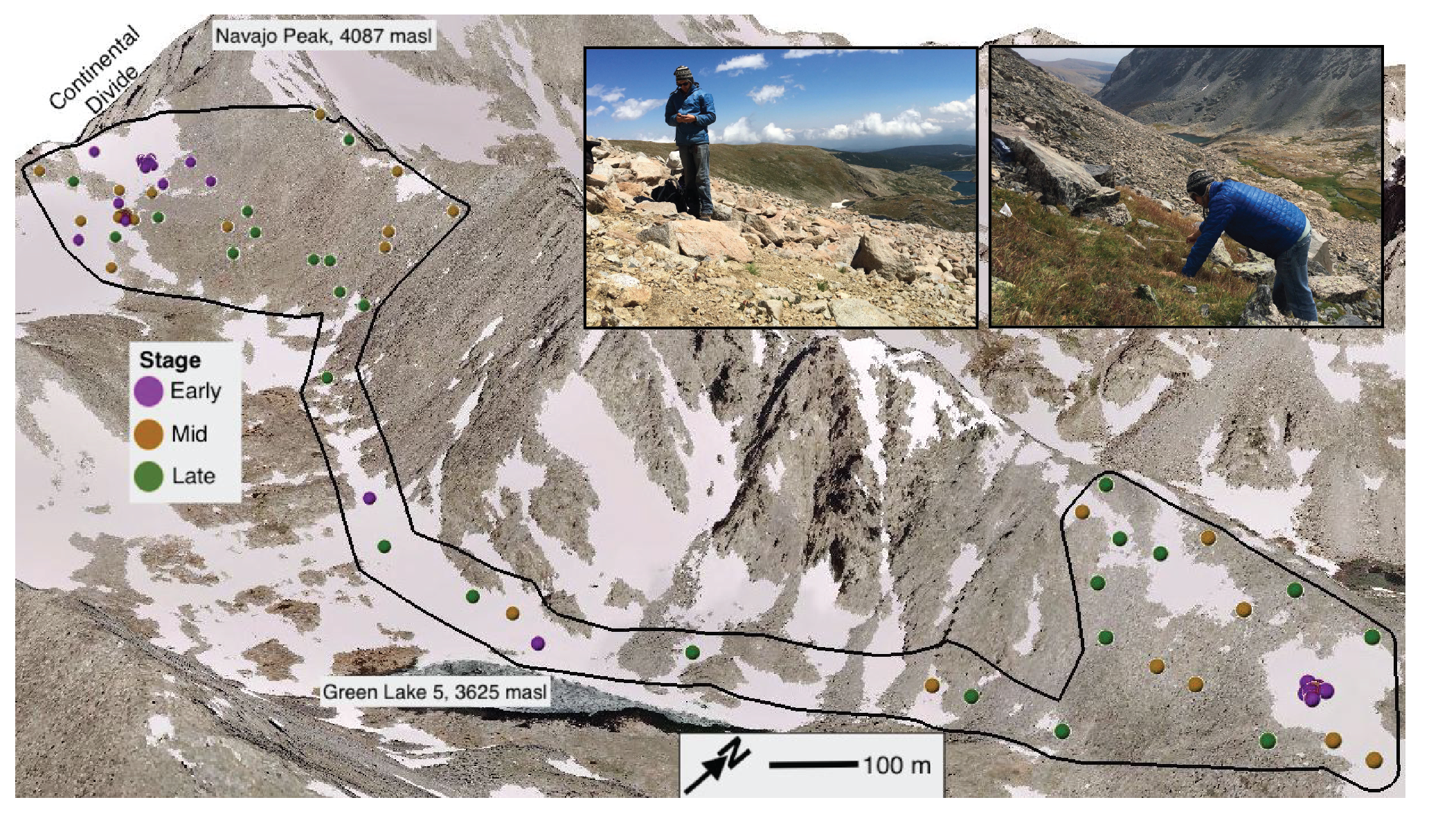


Fig. S2. Rarefaction analysis of microbial community data in Early, Mid, and Late succession plots. Patterns of richness changes over succession shown here are consistent with analyses done with data rarefied to lower sampling depths (Bacteria to 7987, Fungi to 1023, Small Eukaryotes to 871, microfauna to 700, Fig. 1, Fig. S5). Note that the y-axis scales are different for each taxonomic group.

Fig. S3. PCA of plant and environmental variables used to assign successional stage to each plot. Axis 1 was taken to represent successional stage and plots are colored by the successional stage assigned to them.

Fig. S4. Diagnostic plots including residuals vs. fitted values, Q-Q plots, and an example trace plot for early (A, D, G), mid (B, E, H), and late (C, F, I) successional joint distribution modeling analyses.

Fig. S5. Taxonomic (ASV) richness (A-D), proportion rare taxa (E-H), and frequency of microbial taxa (I-L) across succession. Proportion rare taxa was calculated as the proportion of taxa with relative abundances less than 1/S, where S is the mean ASV richness. Significance of successional stage for each group was tested with ANOVA. Values shown are means and standard errors. Note that the y-axis scales are different for each taxonomic group.

Fig. S6. RDAs showing the effect of successional stage on community composition. See Table 1 for statistics.
